# A Clinician and Service User’s Perspective on Managing MS: Pleasure, Purpose, Practice

**DOI:** 10.3389/fpsyg.2020.00709

**Published:** 2020-04-23

**Authors:** Rachael Hunter

**Affiliations:** Department of Psychology, Swansea University, Swansea, United Kingdom

**Keywords:** multiple sclerosis, lifestyle medicine, psychosocial, self-management, behavior modification

## Abstract

There is a growing body of evidence that points to an important role for modification of lifestyle factors and promotion of health-related quality of life in the secondary prevention of disease progression in multiple sclerosis (D’Hooghe et al., 2010; Weiland et al., 2014; Hadgkiss et al., 2015). As a clinical psychologist diagnosed with multiple sclerosis in 2012 I have gained a unique insight into ways in which people living with MS and clinicians can usefully integrate evidence-based lifestyle modifications that enhance self-efficacy and self-management to improve wider psychological and physical health. The framework presented here enables clinicians to engage in salutogenic health promotion by placing value upon the importance of healthy, evidence-based behavior change. Furthermore, the framework provides a structure which can empower and provide guidance for people living with MS on what and how to implement and sustain behavior change and emotional wellbeing in the face of this life-changing diagnosis.

## Introduction

Multiple Sclerosis (MS) is a chronic inflammatory disorder of the central nervous system and it is most frequently diagnosed in young adulthood. With 2.5 million people diagnosed with MS worldwide (Compston and Coles, 2008) and the prevalence increased by 69% between 1996 and 2013 (Rotstein et al., 2018) there is unprecedented demand for support and intervention. There is corresponding growing evidence that modification of lifestyle factors may reduce relapse rate, improve quality of life and potentially slow the course of the disease (Jelinek and Hassed, 2009; D’Hooghe et al., 2010; Jelinek et al., 2013; Weiland et al., 2014; Hadgkiss et al., 2015). If services are to promote and support healthy behavior change among this rapidly growing group of patients, improved methods to help patients and clinicians understand and utilize evidence-based interventions and research to mobilize self-management, are desperately needed. As a clinical psychologist with experience of working in the National Health Service I understand first-hand the challenges faced by services in promoting behavior change. However, it was not until I was diagnosed with MS, in 2012 that I was able to experience for myself the lack of information and encouragement patients receive in relation to making the lifestyle changes research suggests could benefit their symptoms and disease progression. This lack of information and support undoubtedly heightens patient anxiety and leaves them feeling disempowered; both of which contribute to stress-related illness (Theoharides and Cochrane, 2004) and reduce the likelihood of positive behavior change in health interventions (McGrady et al., 2009).

While medication options have increased significantly over the last decade it has been my observation that patients remain poorly informed about the potential role of specific lifestyle risk factors in MS. This is despite a growing body of evidence demonstrating that modification of lifestyle-related risk factors is associated with improved mental and physical health outcomes in MS (Watt et al., 1998; Stuifbergen and Becker, 2001; D’Hooghe et al., 2010; Levin et al., 2014a, b; Taylor et al., 2014; Jelinek et al., 2016; Fitzgerald et al., 2018a, b) and may provide a promising intervention to manage MS progression (D’Hooghe et al., 2010; Li et al., 2010). Specifically, studies have reported that smoking and low vitamin D levels (Coetzee and Thompson, 2017; Hempel et al., 2017), stress-management and mindfulness -based interventions (MBI; Simpson et al., 2014), healthy dietary habits (Hadgkiss et al., 2015) and increasing physical exercise levels (Marck et al., 2014) are all areas in which individuals could make changes to benefit their MS symptoms and quality of life. These findings are in keeping with meta-analyses on health behaviors which have emphasized the central role of diet (Firth et al., 2019) and physical activity (Chekroud et al., 2018) on our psychological and emotional health. Given this growing body of evidence, it is perhaps little wonder that key stakeholders have highlighted the importance of lifestyle as an essential area for future research (Multiple Sclerosis Research of Australia, 2017; Multiple Sclerosis Society of Canada, 2018).

**FIGURE 1:**
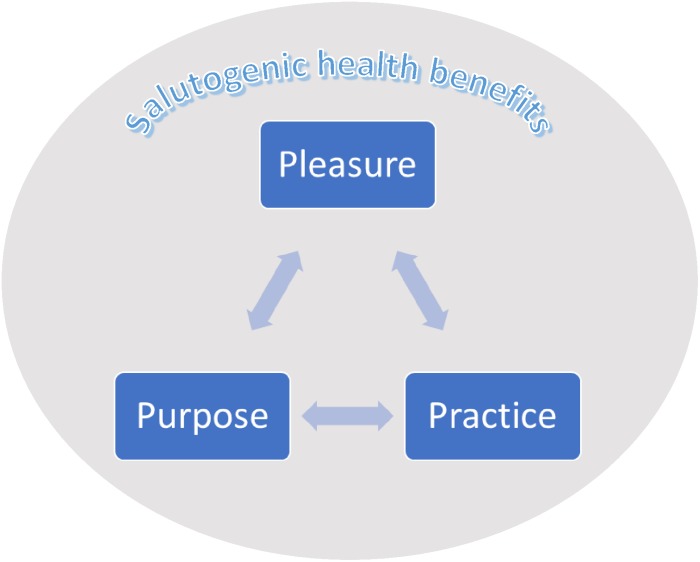
The salutogenic health benefits of the 3P’s framework.

In an era of austerity among clinical services and the growing patient numbers, empowering individuals to make lifestyle modifications seems of paramount importance. Indeed, beneficial self-management programs have emerged in a range of other health conditions such as cancer (Dodd and Miaskowski, 2000) and coronary heart disease (for review see Anderson et al., 2016). However, outside of organized, facilitated psychosocial interventions, which can be costly to services, I have seen that MS patients continue to face barriers to accessing and understanding literature relating to lifestyle medicine and psychoneuroimmunology. As a result, opportunities for patients to understand literature and implement successful behavior change are often limited. My experiences within the MS community and as an academic and clinician, have highlighted three “themes” which may help people living with MS to facilitate and sustain healthy behavior change. These themes (which will be described in this paper and can be seen in Figure 1) may also provide a useful framework through which clinicians can educate and empower their patients. Adopting the accessible approach to health promotion presented below may provide both patients and clinicians with a theoretically and empirically based framework through which healthy behavior change and lifestyle modification may be communicated, implemented and sustained.

### Pleasure

Pleasure and positive affect can co-occur with distress (Folkman and Moskowitz, 2000). Moreover, there is growing evidence that experiencing pleasure and positive emotions is essential to help overcome challenges and improve health-related quality of life (Hildon et al., 2008). Indeed, hedonic theories of wellbeing focus on pleasurable experiences to enhance positive affect (Diener, 1984; Fredrickson, 2001). Most recently, the PERMA model associated with positive psychology and developed by Seligman (2011) highlights the importance of integrating positive emotions and experience for wellbeing. What is more, positive feelings motivate protective human behaviors, enhancing performance at work, strengthening relationships, improving our physical health, encouraging optimism (Kun et al., 2017) which are associated with reduced mortality (Chida and Steptoe, 2008; Diener and Chan, 2011) and create opportunities for enhancing the health and wellbeing of people living with chronic conditions (Mead et al., 2019).

When faced with a life-changing diagnosis many people are triggered to re-evaluate their life and ask, “*what truly makes me happy?”* with many going on to make changes to enable that (Linley and Joseph, 2004). Reprioritisation of values and positive changes in relationships are often reported following trauma and adversity, but notably these also can manifest as increased feelings of intimacy, empathy and greater levels of self-disclosure (Tedeschi and Calhoun, 2004). For many people traumatic and life changing experiences trigger improved psychological functioning (Taylor, 2004) and an increased appreciation of life, setting of new life priorities, increased personal strength, identification of new possibilities, improved intimate relationships, and for some, positive spiritual growth (Tedeschi et al., 1998). Providing accurate information at a time when individuals are open to making radical and positive changes to their life could therefore have far-reaching positive effects for their emotional wellbeing, as well as their physical health. Indeed, the model of Salutogenesis encourages a focus upon, and the benefits of, factors that support health and well-being despite stressors and illness (Antonovsky, 1979, 1987). Essentially, the consultation room offers many opportunities to affect changes that the patient may then take forward outside of that meeting and which can have a positive impact upon their physical and emotional health.

Pleasure is an essential component of the promotion and maintenance of change and should not be undervalued. If we can enjoy the changes we make, we are more likely to persevere (Michie et al., 2008), hereby finding creative and alternative solutions that enhance self-efficacy and esteem as we reflect upon what we have achieved and overcome. From a systemic perspective, engaging in activities which we enjoy also connects us to like-minded people through which we may gain or sustain friendship; making us more likely to sustain behavior change *and* avoid social isolation. With increased likelihood of loneliness in those at risk of social isolation (such as people with long-term health conditions and older people; for reviews see Andersson, 1998; Cacioppo et al., 2000, 2015), and established associations between loneliness and mental health problems (Lasgaard et al., 2016), building these social connections and relationships is protective. They provide a buffering effect, increase levels of perceived support and feelings of self-worth, and encourage maintenance of new, positive habits (Tedeschi and Calhoun, 2004; Cacioppo et al., 2015). Moreover, social connectedness and experiences of pleasure can dramatically effect the construction of social and cultural identities, hereby influencing our understandings of health as well as public health interventions (Coveney and Bunton, 2003).

It is important to pause here and consider that working to integrate experiences of pleasure and positive emotions may take conscious effort – especially for those experiencing pain or disability. And as such, they should be encouraged and fully recognized and supported as valuable to improving wellbeing (Folkman and Moskowitz, 2000). It is important to be mindful that experiencing pleasure is *not* a direct result of an absence of distress – people can experience pleasure in spite of pain or disability; in fact this may enable more existential appreciation of life. Moreover, integrating positive experiences and emotions shape an individual’s subjective experience and encourages adaptation; the kind of flexibility which defines resilience, described here by Pemberton (2015, page 2) as:

“The capacity to remain flexible in our thoughts, feelings, and behaviors when faced by a life disruption, or extended periods of pressure, so that we emerge from difficulty stronger, wiser, and more able.”

As such, integrating positive and pleasurable experiences can help to build resilience and can improve a persons’ quality of life when living with an unpredictable chronic condition such as MS. If we consider the high rates of depression reported among people with MS (lifelong prevalence of approx. 50%; Feinstein, 2011) and the inflammatory nature of stress (Slavich and Irwin, 2014), promoting emotional and positive psychological wellbeing can therefore have a multifaceted impact. For example, contributing to minimizing inflammatory disease activity via the vagus nerve (Kemp et al., 2017a, b), *building protective social networks* and buffering stress (Tedeschi and Calhoun, 2004; Cacioppo et al., 2015).

### Purpose

The stress-inflammation pathway is widely researched (e.g., Segerstrom and Miller, 2004; Steptoe et al., 2007; Denson et al., 2009; Irwin and Cole, 2011) with chronic inflammation associated with a range of autoimmune conditions, cancers and Alzheimer’s disease (Couzin-Frankel, 2010). Cumulative stress and related physiological dysregulation are increasingly regarded as contributing to inflammation, aging and a range of predisposed disease trajectories (Lupien et al., 2009; Juster et al., 2010), even impacting upon gene expression (Zawia et al., 2009; Stankiewicz et al., 2013). As such, person-centered approaches which can contribute to reductions in allostatic load are likely to provide valuable psychosocial interventions for those experiencing stress related illness.

Building a life with the people, purpose and pleasures that are meaningful to you encourages a move away from stressful aspects of your life that may be contributing to chronic stress and perpetuating disease (Slavich and Irwin, 2014). Indeed, interventions that connect to an individual’s purpose or promote meaning and accomplishment have been shown to be protective; increasing well-being and reducing symptoms of depression (Giannopoulos and Vella-Brodrick, 2011; Gander et al., 2013). Antonovsky (1996) argued that “salutogenesis” depends on experiencing a strong “sense of coherence” of which a sense of meaning is essential. Indeed, “sense of coherence” is associated with positive health outcomes, strengthens resilience and encourages a positive subjective state of health (Eriksson and Lindström, 2007). Engaging in activities that are both pleasurable and meaningful also provides opportunities for an individual to experience Csikszentmihalyi’s (2013) concept of “flow”; “the state in which people are so involved in an activity that nothing else seems to matter,” which in turn leads to feelings of happiness (Dodge et al., 2012). Receiving a life-changing diagnosis like MS can lead some individuals to reprioritize and to consider practical changes to their life to make it more fulfilling (Tedeschi and Calhoun, 2006). Such changes in outlook provide opportunity for people to reconnect to the purpose and meaning in their life; resulting in more opportunities for meaningful connection and the positive, protective experience of “flow.” Contact with health care professionals often provides an opportunity to reflect upon the psychosocial impact of diagnosis and life with a chronic condition, and currently opportunities are missed to acknowledge and validate the health benefits of making such changes which would undoubtedly reinforce their impact and empower patients.

### Practice

The notion that stress may trigger disease activity in MS was first considered by Charcot in 1877, and chronic stress is known to significantly affect the function of the immune system (Tausk et al., 2008). The effect of stress on the central nervous system and on the maintenance of the delicate balance between cell-mediated (Th1) and humoral (Th2) immune responses has been studied widely (for review see Chrousos, 2009) with stress reported to activate inflammatory markers and exacerbate symptoms in a range of autoimmune conditions (Theoharides and Cochrane, 2004; Maté, 2011). Given the widely reported efficacy of mindfulness-based interventions (MBI’s) for treating stress, anxiety and recurrent depression in the general population (Goyal et al., 2014) it makes sense that “*MBI’s are effective at improving mental well-being in people with MS”* (Simpson et al., 2014).

Mindfulness may have an effect on acute responses to stress, and a wider impact, by inhibiting underlying consequences of chronic exposure to stress. Simple breathing meditations have the potential to alleviate anxiety and also to help an individual develop self-awareness; supporting better integration of the mind and body (Goyal et al., 2014). Mindfulness and meditation are appealing as psychosocial interventions not least because they have no risks, are free and have originated from 2,500-year-old Buddhist traditions, but also because this legacy is now supported by thousands of peer-reviewed papers (for review see Taylor et al., 2014; Baer, 2015). Key to the efficacy of MBI’s is the cultivation of habitual or daily practice and the setting of intentions to engage in meditation, which, over time, contributes to a more mindful way of being in the world (Bohlmeijer et al., 2010).

Setting intentions to develop new behaviors can provide a useful framework for people living with chronic conditions to work toward. The evidence suggests that engaging in regular meditation and mindfulness practices are likely to benefit people with MS at an emotional and physiological level (Goyal et al., 2014; Senders et al., 2014) even if delivered virtually or remotely (Frontario et al., 2016). MBIs therefore offer an effective, evidence-based and cost-efficient psychosocial intervention which can be used by individuals at little or no costs to them, or to services. As such, there is now no reasonable excuse for clinicians failing to promote low-risk, MBIs as part of a management plan for stress-related and autoimmune illness if we are to improve the wellbeing of people with MS; the majority of which are of working age and many of whom are seeking ways through which they can actively manage their condition. Furthermore, the notion of “practice” and discipline is a useful notion which can then be extended to other health lifestyle changes – for example, changes to exercise regime or diet. Setting such intentions encourages people to plan how they will implement such changes and helps them to consider how they will overcome barriers and frustrations (Chapman et al., 2009).

### Discussion

The framework presented here provides a useful structure for people living with MS, and clinicians working with them, to approach secondary prevention. Advances in medical treatment over the last decade have been hugely positive for the MS community, however, shifts in incorporating evidence relating to lifestyle modifications have lagged behind (Jelinek and Hassed, 2009). This is despite the known benefits of encouraging healthy behavior change (Hadgkiss et al., 2013) and patients demands for non-drug interventions in MS (Leong et al., 2009). The growing numbers of people being diagnosed with MS point to an urgent need to incorporate this information and provide more specific guidance and encouragement regarding self-management. However, as a health professional I know it can be challenging for clinicians to be aware of and/or summarize the rapidly evolving evidence-base, and even harder to develop individualized plans for patients. Austerity measures, alongside people’s widening access to health information online provide opportunities for people to autonomously implement healthy changes to their life and clinicians can support and encourage this by using this simple, evidence-based framework.

Communication between “patient” and doctor provides “an important role in educating and motivating patients to take appropriate actions and assist in shared decision-making for improved health outcomes” (Teutsch, 2003). The dissemination of knowledge needs to be enhanced and research evidence needs to be made more accessible (Harzing and Adler, 2016). The promotion of healthy behavior change should not be avoided by clinicians and, based on the empirical knowledge available, to do so neglects the very premise of health care. Indeed disseminating psychological knowledge is a core purpose of the role of a clinical psychologist (British Psychological Society, Biddle et al., 1992) and as such, many clinicians already have a range of skills in their “tool kit” to facilitate the propagation of this information. Patient-doctor communication can play an important role in educating and motivating patients to take appropriate actions and assist in shared decision-making for improved health outcomes. Key health care providers such as neurologists and specialist nurses have the opportunity and potential to empower many patients with MS to take more control of their health by highlighting the benefits of self-efficacy and value of lifestyle modification during consultations – such interactions present important opportunities to acknowledge and place value on the role of specific lifestyle factors such as vitamin D supplementation, exercise, stress management and nutrition. Access to psychological support and targeted health promotion is often inhibited by the limited resources available to clinicians and few MS services are able to provide specialist psychological support to all their patients. As such, the important role that neurologists and nurses can play in promoting and facilitating healthy behavior change in patients must be acknowledged and enhanced. My experience as a patient and professional has shown me that many MS specialist clinicians feel unnecessary discomfort at recommending specific secondary prevention or “lifestyle” interventions and, as a result, avoid discussing anything except the most rudimentary lifestyle factors. This ambiguity can leave patients feeling like they do not have the support of their medical team in making lifestyle and behavior modifications, and/or that such changes are not worthwhile. Opportunities for health promotion are consequently missed as a result of the clinicians reticence and this can have implications for the patients decisions to engage more actively in self-management.

Salutogenic approaches such as the one described here while not widespread are not entirely novel. They have been used in the development of health promotion models (Eriksson and Lindström, 2006), the development of therapeutic practice in health care (Pelikan, 2017), and the design of health-care environments (Wallerstein, 1992; Dilani, 2008). Of particular value in this case is the concept of “sense of coherence” (SOC) which refers to collective coping and acknowledges the ability to mobilize resources in the face of challenge (Taylor, 2004). In particular, SOC highlights the ability for people to understand what happens to them, the extent to which they are able to manage the situation on their own or with the support of community, and the ability to find meaning in the situation (Antonovsky, 1998; Eriksson and Lindström, 2006). Considered alongside the established evidence that interventions which modify self-efficacy are effective in promoting health behavior change (Sheeran et al., 2016) and the growing numbers of people diagnosed with MS (Rotstein et al., 2018), new approaches to management are essential.

The simple framework described here; “The 3 P’s of Secondary Prevention,” offers a risk-free structure from which clinicians can encourage and support their patients in evidence-based lifestyle modifications (D’Hooghe et al., 2010; Weiland et al., 2014; Hadgkiss et al., 2015). Moreover, such empowerment carries known health benefits (Wallerstein, 2007) and should not be “*restricted to some disease and treatment-related outcomes, but should be discussed and negotiated with every patient*” (Aujoulat et al., 2007). Engaging people with MS as potential collaborators in their care will not only foster greater patient satisfaction and improve adherence (Martin et al., 2005), it also allows for enhanced understanding of the individuals specific socio-cultural context (Kreps, 2006) and can have potential benefits for individual wellbeing and wider service efficacy (Jahng et al., 2005). However, it is important to consider that the framework is not without its weaknesses. It may, for example, prove difficult for clinicians to feel confidant if patients ask for further information about areas in which the clinician does not feel knowledgeable. There may also be limitations of time within patient consultations which make discussions of secondary prevention difficult. Despite these considerations it is important to hold in mind that for all those patients who are able to independently seek out and decipher up-to-date research evidence and make confidant autonomous decisions about their health care, there are many others for whom this is simply not possible. Clinicians can play an important role in mobilizing patients to engage in salutogenic, health promoting behavior change by making these simple changes in *what* and *how* they communicate with patients. Encouraging patients to take an active role in the management of their MS, which is a life-long chronic condition can have far-reaching positive benefits for their quality of life, physical and emotional wellbeing (Bogosian et al., 2015). If more MS specialist services are able to implement such an approach they may well provide the medical ‘approval’ the most at-risk patients feel they require to explore and truly engage with and implement such behavior modifications. This may, in the long-term, have the potential to radically change the face of self-management and the quality of life of people living with MS.

## Author Contributions

The author confirms being the sole contributor of this work and has approved it for publication.

## Conflict of Interest

The author declares that the research was conducted in the absence of any commercial or financial relationships that could be construed as a potential conflict of interest. The handling Editor declared a shared affiliation, though no other collaboration, with the author RH at the time of review.
